# The Medical Student’s Vade Mecum; a Compendium of Anatomy, Physiology, Chemistry, Materia Medica and Pharmacy, Surgery, Obstetrics, Practice of Medicine, Diseases of the Skin, Poisons, &c. &c.

**Published:** 1852-07

**Authors:** 


*The Medical Student's Vade Mecum: a Compendium of Anatomy, Physiology, Chemistry, Materia Medica and Pharmacy, Surgery, Obstetrics, Practice of Medicine, Diseases of the Skin, Poisons, &c. &c.* By GEORGE MENDENHALL, M.D., Third edition. Philadelphia, Lindsay & Blakiston, 1852.

Of the third edition, lately issued, of this useful work, we have great pleasure in echoing the generally expressed favorable opinion of the medical press. It is, in every respect, well arranged and well executed, and deserves the popularity which has been established by "the rapid sale of the two former editions." As regards the form of "question and answer," by which this work is characterised, our opinion has been that it is needlessly cumbersome, occupying space that might be better employed. We presume, however, from its having been retained here, through three editions, that it is not unacceptable to the class for whom it is intended.

